# Hydroxychloroquine Mitigates Cytokine Storm and Prevents Critical Illness Neuromyopathy in a Rat Sepsis Model

**DOI:** 10.3390/medicina60111791

**Published:** 2024-11-01

**Authors:** Elif Eygi, Onur Kucuk, Semih Aydemir, Merve Atilgan, Recep Dokuyucu, Oytun Erbas

**Affiliations:** 1 Department of Anesthesiology and Reanimation, Gaziantep City Training and Research Hospital, Gaziantep 27470, Turkey; drelifeygi6@gmail.com; 2 Department of Anesthesiology and Reanimation, Ankara Atatürk Sanatorium Training and Research Hospital, University of Health Sciences, Ankara 06000, Turkey; dr.okucuk@gmail.com; 3 Department of Anesthesiology and Reanimation, Yenimahalle Training and Research Hospital, University of Yıldırım Beyazit, Ankara 06370, Turkey; drsemihaydemir@gmail.com; 4 Department of Pediatric Surgery, School of Medicine, Necmettin Erbakan University, Konya 42140, Turkey; atasmerve66@gmail.com; 5 Department of Physiology, Medical Specialization Training Center (TUSMER), Ankara 06230, Turkey; 6 Department of Physiology, School of Medicine, Demiroglu Bilim University, Istanbul 34570, Turkey; oytunerbas2012@gmail.com

**Keywords:** critical illness neuromyopathy, hydroxychloroquine, interleukin-6, malondialdehyde, tumor necrosis factor-alpha, muscle action potential

## Abstract

*Background and Objectives:* It is known that critical illness and associated neuromuscular problems begin to appear in patients hospitalized in the intensive care unit (ICU) for more than a week. The goal of this study was to research the role of hydroxychloroquine (HCQ) in the treatment of cytokine storm and critical illness neuromyopathy (CINM) in a rat sepsis model. *Materials and Methods:* Rats were assigned into three groups, and a feces intraperitoneal-injection group (FIP) procedure was carried out on 30 rats to induce a model of sepsis for critical illness polyneuromyopathy (CINM). The study groups were as follows: Group 1: control (nonoperative and orally fed control, *n* = 10), Group 2: FIP with 0.9% NaCl saline was given as 1 mL/kg/day by oral gavage (*n* = 10), and Group 3: FIP with 10 mg/kg/day of hydroxychloroquine (Plaquenil 200 mg) administered by oral gavage (*n* = 10). Electrophysiological recordings (EMG) were conducted six days after surgery. EMG was carried out three times on the right sciatic nerve, which was stimulated with supramaximal intensity utilizing a bipolar needle electrode at the sciatic notch. Tumor necrosis factor-alpha (TNF-α), malondialdehyde (MDA), lactic acid levels, and interleukin-6 (IL-6) were evaluated. *Results:* In terms of TNF-α, MDA, lactic acid levels, and IL-6, there was a statistically significant decrease in the CINM + 10 mg/kg HCQ group compared to the CINM and saline group (*p* < 0.0001, *p* < 0.05, *p* < 0.05, and *p* < 0.05, respectively). Compound muscle action potentials (CMAPs) latency and duration were decreased in the CINM + 10 mg/kg HCQ group compared to other groups (*p* < 0.01 and *p* < 0.001). However, CMAP amplitude was significantly higher in the CINM + 10 mg/kg HCQ group unlike the CINM and saline group (*p* < 0.001). *Conclusions:* This is the first study to demonstrate the effects of HCQ on CINM in a rat model of sepsis. The findings of our research suggest that hydroxychloroquine may be used as a potential therapeutic agent in the treatment of sepsis. Hydroxychloroquine may have an important effect in the pathogenesis of sepsis-associated CINM by reducing cytokine production and oxidative stress.

## 1. Introduction

It is known that critical illness and associated neuromuscular problems begin to appear in patients hospitalized in the intensive care unit (ICU) for more than a week [1,2]. Critical illness, which manifests itself with loss of strength in the extremities and respiratory muscles and the accompanying difficulty in weaning from mechanical ventilation, may be in the form of polyneuropathy, myopathy, or a combination of these. These diseases, whose importance has been increasingly emphasized by systematic studies conducted in recent years, present some diagnostic difficulties, although they are common. However, they are associated with high rates of mortality and morbidity. So, its course is directly linked to the underlying disease [3,4].

In patients in the ICU, loss of strength may occur primarily as a result of a disease of the neuromuscular region such as myasthenia gravis, inflammatory myopathy or polyradiculoneuropathy, or secondary to sepsis, multiple organ failure, trauma, or the use of certain medications [1,2,3,4]. Being able to make this distinction is important to making inferences about the course of the disease and survival and determining appropriate treatment strategies.

The currently accepted view of critical illness neuropathy (CIN) is that this disease is strongly associated with systemic inflammatory response syndrome, sepsis, or multiple organ failure. It is thought that the microcirculation disorder seen in all organs during these severe clinical conditions also affects the peripheral nerves and causes polyneuropathy [1,4,5]. According to this proposed model, in the presence of systemic inflammatory response syndrome, multiple organ failure, or sepsis, antigen-presenting cells are triggered and secrete pro-inflammatory cytokines such as interleukin-1, interleukin-12, and TNF-α. As a result, helper T cells, neutrophils, macrophages, and monocytes are activated [6].

It is reported that one of the important causes of weakness in intensive care patients is critical illness myopathy (CIM) [5]. The pathogenesis of CIM is mitochondrial dysfunction, which develops especially secondary to sepsis, and the energy failure caused by oxidative stress and other factors triggered by this, and the damage caused by this [7].

Looking at the studies in the literature, there is no specific treatment for both CIM and CIN. Since the two diseases occur together in sepsis, it is called critical illness polyneuromyopathy (CINM). Treatment should be directed at the underlying disease, that is, sepsis. Early recognition of sepsis and targeted treatment both prevent the development of CINM and increase the treatability of existing CINM. The disease should be diagnosed at an early stage, and the development of multiple organ failure or irreversible microvascular circulatory disorder should be prevented by applying appropriate antibiotics, supportive treatment, and necessary surgical interventions [6,8].

Chloroquine and hydroxychloroquine (HCQ) are well-known antimalarial drugs that have been extensively used for decades, not only in the prevention and treatment of malaria but also in the management of autoimmune and rheumatic diseases such as systemic lupus erythematosus and rheumatoid arthritis. These drugs exhibit their effects through multiple mechanisms, acting as immunomodulators [9,10]. They modulate the host immune response by inhibiting cytokine production (such as IL-6 and TNF-α), reducing autophagy, and impairing lysosomal activity in host cells. These actions result in the suppression of inflammatory processes, which is why they are beneficial in treating autoimmune diseases [10,11]. Additionally, both chloroquine and HCQ increase the pH within lysosomes and endosomes, disrupting cellular pathways that are crucial for the activation of immune cells and the replication of intracellular pathogens. Clinically, HCQ is often preferred due to its improved safety profile, particularly concerning the reduced risk of retinal toxicity compared to chloroquine [9,12,13].

The goal of this study was to research the role of HCQ in the treatment of cytokine storm and CINM in a rat model of sepsis.

## 2. Materials and Methods

### 2.1. Study Design and Study Population

In this research, 30 mature Wistar albino rats, 12 weeks old and weighing between 300 and 350 g, were utilized. The rats were sourced from the animal laboratory of science university. The rats were divided into three groups. The feces intraperitoneal-injection (FIP) protocol was conducted on 30 rats to create a sepsis pattern for studying CINM. The FIP model was created following the methodology described by Shrum et al. and Tyml et al. [14,15]. Feces were gathered and mixed with saline to create a feces–saline solution. This solution was injected intraperitoneally into the rats at a dose of 1 g per kilogram of body weight. Six rats did not survive the second day after the model was created and were excluded from the research.

The study groups were as follows:

Group 1: Normal (nonoperative and orally fed control, *n* = 10);

Group 2: FIP with 0.9% NaCl saline was given as 1 mL/kg/day by oral gavage (*n* = 10);

Group 3: FIP with 10 mg/kg/day of hydroxychloroquine (Plaquenil 200 mg, sanofi aventis) administered by oral gavage (*n* = 10).

The dosage of 10 mg/kg/day of HCQ used in this study was determined based on previous animal studies that investigated the efficacy and safety of HCQ in experimental models of inflammation and sepsis [16,17]. All treatments were given over a two-day period. At the conclusion of the second day, electromyography (EMG) was conducted, and samples were gathered. For the surgical procedure, the rats were anesthetized with an i.p. injection of xylazine hydrochloride (40 mg/kg) and ketamine hydrochloride (80 mg/kg). The animals were then euthanized by high-dose anesthesia (xylazine hydrochloride (80 mg/kg) and ketamine hydrochloride (100 mg/kg)).

### 2.2. Measurements

Plasma levels of IL-6 and TNF-α were measured using enzyme-linked immunosorbent assay (ELISA) kits. Specifically, IL-6 levels were quantified using the Rat IL-6 ELISA kit (Biosciences, San Diego, CA, USA), and TNF-α levels were measured using the Rat TNF-α ELISA kit (Abcam, Cambridge, MA, USA). Both assays were performed following the manufacturers’ protocols. The procedure involved collecting blood samples from the rats, which were centrifuged at 3000 rpm for 10 min to separate plasma. The plasma samples were then diluted appropriately, and the ELISA assays were performed in 96-well plates coated with specific antibodies against IL-6 and TNF-α. After the incubation and washing steps, the bound cytokines were detected using a secondary antibody conjugated with horseradish peroxidase (HRP). The reaction was developed using a TMB (3,3′,5,5′-tetramethylbenzidine) substrate, and the absorbance was read at 450 nm with a microplate reader. Standard curves for both IL-6 and TNF-α were generated using known concentrations, allowing us to determine the cytokine levels in each sample.

Lactic acid levels were measured using a gas analyzer (e.g., Radiometer ABL800, Radiometer Medical ApS, Copenhagen, Denmark), which is a reliable tool for evaluating metabolic status. Blood samples were drawn from the animals and immediately analyzed to determine lactic acid concentrations. Lactic acid is a critical biomarker in sepsis, indicating tissue hypoxia and anaerobic metabolism. The gas analyzer works by analyzing whole blood or plasma samples and provides results for lactic acid concentration in millimoles per liter (mmol/L). The rapid and precise quantification of lactic acid in this manner allowed us to assess the metabolic impact of sepsis and the potential mitigating effects of hydroxychloroquine on metabolic derangements.

Lipid peroxidation in plasma samples was evaluated by determining malondialdehyde (MDA) levels, which are indicative of thiobarbituric acid reactive substances (TBARSs). The method used for MDA determination involved the reaction between MDA and thiobarbituric acid (TBA) under acidic and high-temperature conditions, forming a pink-colored MDA-TBA complex. Blood samples were collected from the rats, and plasma was separated as described above. Plasma samples were mixed with TBA reagent and incubated in a boiling water bath for 10 min. After cooling, the samples were centrifuged at 4000 rpm for 10 min to separate the precipitated proteins. The absorbance of the supernatant was then measured at 532 nm using a spectrophotometer. The MDA concentration was calculated using an extinction coefficient and expressed in nanomoles per milliliter (nmol/mL). MDA is widely used as a marker of oxidative stress, and its levels reflect the degree of lipid peroxidation, which is closely related to the extent of oxidative damage in tissues.

### 2.3. Electrophysiological Recordings

Electrophysiological recordings were conducted using electromyography (EMG) to evaluate the neuromuscular function in all groups. These recordings were performed six days after the surgical procedure to assess the impact of sepsis and HCQ treatment on neuromuscular transmission, particularly focusing on CINM.

The right sciatic nerve was chosen for stimulation due to its accessibility and well-established use in rodent models of neuromuscular dysfunction. To ensure consistent stimulation, the sciatic nerve was stimulated using a bipolar needle electrode (BIOPAC Systems, Goleta, CA, USA) placed at the sciatic notch. The stimulation intensity was adjusted to supramaximal levels, meaning that the intensity exceeded the threshold necessary to activate all motor units within the nerve, ensuring that the recorded responses reflected the full neuromuscular output. This was crucial for obtaining reliable compound muscle action potentials (CMAPs), which serve as a direct indicator of neuromuscular function.

The recording of CMAPs was performed on the second and third interosseous muscles of the hind limb, as these muscles are innervated by the sciatic nerve, making them suitable for detecting motor unit responses. Unipolar platinum electrodes were used to capture the muscle action potentials, which were generated in response to nerve stimulation. Platinum electrodes were chosen for their high sensitivity and low impedance, providing accurate recordings of small electrical potentials.

Each EMG session involved three separate stimulations, with a focus on evaluating the amplitude, duration, and latency of the CMAPs. These parameters are critical in assessing neuromuscular integrity:

- Amplitude of the CMAPs represents the number of muscle fibers that are successfully activated in response to nerve stimulation. A reduction in amplitude could indicate axonal damage or impaired neuromuscular transmission, both of which are hallmarks of CINM.
- Duration of the CMAPs reflects the time span during which the muscle fibers are activated. Prolonged durations may indicate impaired conduction or demyelination within the nerve.
- Latency measures the time delay between the onset of nerve stimulation and the initiation of muscle contraction. Increased latency may suggest nerve conduction slowing, which is commonly associated with inflammatory or metabolic changes affecting the nerve.

**Figure 1:**
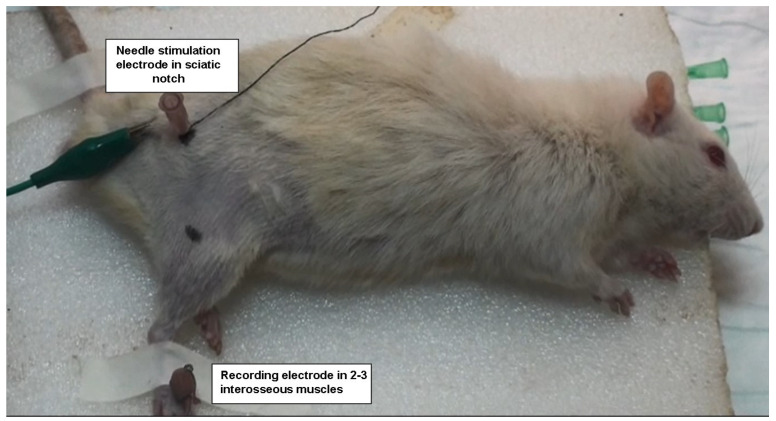
Electrophysiological recordings or electromyography (EMG) measurements.

The data from these recordings were processed and analyzed using Biopac Student Lab software version 5.0 (BIOPAC Systems, USA), a specialized software designed for educational and research purposes in the field of electrophysiology. The software allowed for real-time data capture, display, and subsequent analysis of the recorded CMAPs, providing precise measurements of the amplitude, duration, and latency of each signal. These primary parameters were used to evaluate the effects of hydroxychloroquine on neuromuscular function in the context of sepsis and CINM (Figure 1).

We acknowledge that the potential influence of electrolyte imbalances (e.g., hypocalcemia, hypomagnesemia, hypoglycemia) was not evaluated in this study. These imbalances can affect neuromuscular excitability and influence the EMG results. Future studies should include monitoring of serum electrolytes and glucose to account for their possible effects on neuromuscular function and electrophysiological recordings.

### 2.4. Statistical Analysis

For statistical analysis, SPSS version 27.0 (IBM Corp., Armonk, NY, USA) was performed. For parametric variables and comparisons between groups, an ANOVA test and Student’s *t*-test were used. The Mann–Whitney U test was used for nonparametric variables. To differentiate between parametric and nonparametric variables, the Shapiro–Wilk test was performed. Results are presented as mean ± SEM. A *p*-value < 0.05 was regarded as statistically significant.

## 3. Results

A comparison of inflammatory markers between groups is shown in Table 1. In terms of TNF-α, MDA, IL-6, and lactic acid levels, there was a statistically significant increase in the CINM and saline group compared to the control group (*p* < 0.0001, *p* < 0.0001, *p* < 0.01, and *p* < 0.0001, respectively). However, in terms of MDA, TNF-α, lactic acid, and IL-6, there was a statistically significant decrease in the CINM and 10 mg/kg HCQ group compared to the CINM and saline group (*p* < 0.05, *p* < 0.0001, *p* < 0.05, and *p* < 0.05, respectively) (Table 1).

**Figure 2:**
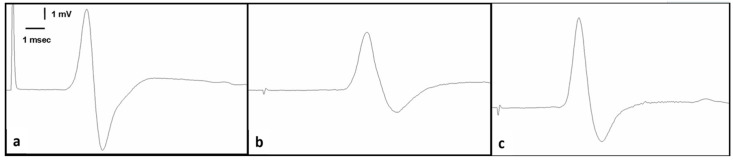
Samples of compound muscle action potential (CMAP) recorded from (**a**) normal group, (**b**) feces intraperitoneal-injection (FIP) and saline group, and (**c**) FIP and 10 mg/kg hydroxychloroquine group.

A comparison of EMG values between groups is shown in Table 2. In terms of CMAP latency and CMAP duration values, there was a statistically significant increase in the CINM and saline group compared to the control group (*p* < 0.01, *p* < 0.01, and *p* < 0.0001, respectively). However, in terms of CMAP latency and CMAP duration values, there was a statistically significant decrease in the CINM and 10 mg/kg HCQ group compared to the CINM and saline group (*p* < 0.01, *p* < 0.01, and *p* < 0.0001, respectively). In terms of CMAP amplitude values, there was a statistically significant decrease in the CINM and saline group compared to the control group (*p* < 0.0001). However, in terms of CMAP amplitude, values decreased in the CINM and 10 mg/kg HCQ group compared to the CINM and saline group (*p* < 0.001) (Table 2, Figure 2).

## 4. Discussion

This is the first study to show the effects of HCQ on CINM in a rat sepsis protocol. The findings of this study demonstrate the protective effects of HCQ on cytokine storm and CINM in a rat sepsis protocol. These results align with the existing literature that highlights the pathophysiological mechanisms and potential therapeutic strategies for managing sepsis-induced complications.

CINM is a condition frequently seen in patients hospitalized in the ICU for a long time. It has been widely discussed in the literature that CINM develops secondary to sepsis and is directly related to the severity of sepsis [18,19,20,21,22]. Erbas et al. reported that the imbalance between inflammatory processes and oxidative and antioxidative states contributes significantly to the development of CIN. Their research demonstrated that administering oxytocin and melatonin to rats markedly reduced EMG amendments and decreased oxidative stress and TNF-α levels in cases of CIN. Consequently, they suggest that both oxytocin and melatonin might offer protective benefits against sepsis-induced polyneuropathy in critically ill patients [21]. In a study by Solmaz et al., it was reported that lacosamide might have valuable effects on early sepsis-induced critical illness neuropathy due to its potential anti-inflammatory properties and ability to inhibit lipid peroxidation. Their findings revealed that plasma MDA and TNF-alpha levels were lower in rats administrated with 20 mg/kg and 40 mg/kg of lacosamide in cases of CIN [22]. Solmaz et al. reported that papaverine exhibited neuroprotective effects in a rat model of CIN. Treatment with papaverine resulted in reduced levels of TNF-α, MDA, CRP, lactic acid, and IL-6 relative to the CIN group. They suggest that papaverine, owing to its antioxidant and anti-inflammatory features, has significant neuroprotective effects [20]. Silistre et al. showed that the remedial role of ascorbic acid on CIN was demonstrated using a rodent sepsis model. Treatment with ascorbic acid significantly decreased plasma levels of IL-6, MDA, and TNF-α in cases of CIN. The researchers concluded that ascorbic acid exhibited notable antioxidant and anti-inflammatory features, leading to its ameliorative effects in the rat model of CIN [19]. In our study, it was observed that HCQ treatment drastically decreased the levels of IL-6 and TNF-α in sepsis-induced rats. This finding supports the information in the literature that cytokine storm plays a significant role in CINM pathophysiology.

Hydroxychloroquine (HCQ) is a drug known for its antimalarial and anti-inflammatory effects. So, the use of HCQ in the treatment of various infections and inflammatory diseases has increased in recent years [23,24]. Recent studies have also explored the antioxidative effects of HCQ, particularly its ability to lower MDA levels. Malondialdehyde is a byproduct of lipid peroxidation and serves as a reliable marker of oxidative stress. Research indicates that HCQ can reduce oxidative stress by inhibiting lipid peroxidation, thereby lowering MDA levels in patients with inflammatory diseases. This antioxidative effect contributes to the overall protective benefits of HCQ in managing chronic inflammation and preventing tissue damage [25]. In a study on rheumatoid arthritis patients, HCQ was found to decrease oxidative stress markers, including MDA, indicating its potential to reduce oxidative damage associated with chronic inflammation [24]. Similarly, in patients with systemic lupus erythematosus, HCQ’s ability to modulate oxidative stress markers has been related with optimized clinical outcomes and decreased disease activity [23]. In our study, it was found that HCQ treatment reduced lipid peroxidation by reducing MDA and lactic acid levels in sepsis-induced rats. This is consistent with the findings in the literature that hydroxychloroquine reduces oxidative stress.

The EMG results revealed that rats given hydroxychloroquine showed significant improvements in CMAP amplitude and duration. These findings provide evidence that HCQ improves neuromuscular transmission [26]. It has been reported in the literature that similar electrophysiological parameters have been examined in animal models of sepsis and that various treatment approaches have positive effects on these parameters [19,20,21,22]. In a study by Erbas et al., the treatment of melatonin and oxytocin to rats drastically decreased the EMG amendments. So, they stated that both melatonin and oxytocin may have favorable effects to protect from CIN [21]. In a study by Solmaz et al., it was reported that both CIN plus lacosamide (20 and 40 mg/kg) groups had better CMAP variables but only compared to the CIN group [22]. In another study by Solmaz et al., it was stated that CMAP variables (latency and duration) were shorter in the group given only papaverine (40 mg/kg) relative to the CIN [20]. In a study by Silistre et al., they stated that a considerable increase in CMAP amplitude and a notable decrease in CMAP latency were evaluated in the CIN plus ascorbic acid treatment group relative to the CIN group [19]. In our study, while latency and duration values of CMAPs increased significantly in the CINM group relative to the control group, CMAP amplitude values were significantly lower. However, CMAP latency and duration were significantly decreased, while CMAP amplitude was significantly higher in the CINM + 10 mg/kg HCQ group relative to the CINM + saline group.

### Limitations of This Study

There were some limitations in the current study. We aimed to assess the effect of HCQ on oxidative properties and inflammation by measuring IL-6, MDA, TNF-α, and lactic acid. However, we were unable to identify the specific mechanisms through which HCQ exerts these effects, and we could not conclusively determine whether HCQ possesses antioxidant properties.

In addition to the limitations mentioned earlier, we also recognize that the homogeneity of the groups, individual inflammatory responses, sepsis stages, and electrolyte imbalances (such as hypocalcemia, hypomagnesemia) were not accounted for in this study [27,28]. These factors can influence neuromuscular function and potentially alter the EMG results. Future studies should address these variables to provide a more comprehensive understanding of the neuromuscular effects in sepsis-related critical illness neuromyopathy.

## 5. Conclusions

In conclusion, positive effects of hydroxychloroquine were observed on sepsis and sepsis-related CINM in our study. The findings of our study suggest that hydroxychloroquine may be used as a potential therapeutic agent in the treatment of sepsis. Hydroxychloroquine targets mechanisms that play a critical role in the pathogenesis of sepsis-associated CINM by reducing cytokine production and oxidative stress. Other studies in the literature also support such effects of hydroxychloroquine and highlight its potential for use in clinical applications. Future studies are important to further solidify the place of this drug in the treatment of sepsis.

## Figures and Tables

**Table 1:** Comparison of inflammatory markers between groups.

CMAP Variables	Control	CINM + Saline	CINM + 10 mg/kg HCQ
MDA (nM)	71.04 ± 6.1	221.5 ± 13.7 **	115.8 ± 11.3 #
TNF-α (pg/mL)	15.2 ± 2.35	248.1 ± 16.4 **	82.3 ± 6.29 ##
IL-6 (pg/mL)	21.4 ± 5.6	82.3 ± 13.08 *	34.9 ± 7.5 #
Lactic acid (mmol/L)	1.3 ± 0.38	7.02 ± 0.94 **	2.8 ± 0.65 #

CINM: critical illness polyneuromyopathy, HCQ: hydroxychloroquine, MDA: malondialdehyde, TNF-α: tumor necrosis factor-alpha, IL-6: interleukin-6. Results are presented as mean ± SEM. Statistical analyses were conducted using one-way ANOVA. The following significance levels were used: * *p* < 0.01, ** *p* < 0.0001 compared to normal groups; # *p* < 0.05, ## *p* < 0.0001 compared to the feces intraperitoneal-injection (FIP) and saline group.

**Table 2:** Comparison of electromyography (EMG) values between groups.

CMAP Variables	Control	CINM + Saline	CINM + 10 mg/kg HCQ
Latency (ms)	2.39 ± 0.09	3.08 ± 0.15 *	2.56 ± 0.05 #
Duration (ms)	2.71 ± 0.14	3.37 ± 0.25 *	2.88 ± 0.12 #
Amplitude (mV)	12.8 ± 0.66	6.34 ± 0.55 **	10.05 ± 0.54 ##

CMAP: compound muscle action potential, CINM: critical illness polyneuromyopathy, HCQ: hydroxychloroquine. Results are presented as mean ± SEM. Statistical analyses were conducted using a one-way ANOVA. The following significance levels were used: * *p* < 0.01, ** *p* < 0.0001 compared to normal groups; # *p* < 0.05, ## *p* < 0.0001 compared to the feces intraperitoneal-injection (FIP) and saline group.

## Data Availability

Data are available upon request to the corresponding author.
